# Beneficial and Detrimental Effects of Uric Acid on Alzheimer’s Disease

**DOI:** 10.2174/011570159X349365250128072146

**Published:** 2025-02-18

**Authors:** Olga Tovchiga, Iwona Inkielewicz-Stepniak

**Affiliations:** 1 Department of Pharmaceutical Pathophysiology, Faculty of Pharmacy, Medical University of Gdansk, Debinki 7, Building 27, Gdansk, PL-80211, Poland

**Keywords:** Uric acid, neuroprotection, Alzheimer’s disease, modulation of the protective effect, xanthine oxidase interrelationship, amyloid-β pathway

## Abstract

The interconnection between brain function and hyperuricemia remains controversial since the available evidence indicates both the potent neuroprotective role of uric and its negative cardiovascular and metabolic effects, possible prooxidant activity. A mixed (protective and risk) effect of uric acid (UA) on neurological disorders was assumed. Among the neurodegenerative diseases, Alzheimer’s disease remains the most prevalent, causes disability, and lacks highly effective treatments. Therefore, this review aims to delineate the beneficial and detrimental effects of uric acid on Alzheimer’s disease (AD). This can not only facilitate estimating the benefits and risks of urate-lowering or urate-increasing interventions in different conditions but also can enhance understanding of the molecular pathways associated with the protective role of uric acid, leading to the identification of new therapeutic targets for neuroprotection. Firstly, we addressed interconnections between UA and AD in different patients and population subgroups. Secondly, we analysed which differences can arise at the level of uric acid transport to the brain, its influence on blood-brain barrier (BBB), and its presence in brain tissue and cerebrospinal fluid. Such aspects as xanthine oxidase interrelationship with the risk of cognitive impairment was elucidated, as well as the unexpected interconnection between uric acid exchange and the cholinergic system. Finally, an analysis was done of the beneficial and detrimental effects of uric acid on such targets of Alzheimer’s disease pathogenesis as the amyloid-β pathway, proinflammatory markers, peroxynitrite scavenging, and other aspects of prooxidant-antioxidant status.

## 1 INTRODUCTION

The remarkable interconnection between hyperuricemia and high cognitive function is an important factor in human evolution and societal development [1, 2] and remains an inspiring and challenging issue. New insights in this field are possible due to the development of modern methods.

Within the broad biological problem of the influence of high levels of uric acid (UA) on the central nervous system (CNS), the possible counteraction to dementia development and, in particular, to Alzheimer’s disease (AD) has garnered significant attention. In addition to the increase in motivation for active work and realization of an individuum’s abilities contributing to the highest achievements and genius formation [2], the protective influence of high UA levels can be realized at the other end of the spectrum, namely, in the case of dementia.

However, the role of hyperuricemia as a risk factor for “diseases of civilization,” its negative cardiovascular effects, its involvement in inflammatory processes in the context of gout development, and the possibility of the conversion of its antioxidant effect to a prooxidant [3-5] cannot be neglected. The links between UA and fructose metabolism, as well as between UA and salt hypersensitivity, sodium retention, and maintenance of blood pressure, have been profoundly investigated in the works of Prof. R.J. Johnson and collaborators [6-8].

The concept of Johnson *et al*. (2009) on the activatory role of UA in changing the behavioural responses of animals upon the transition to procuring food (“the foraging response”) [6] was considered one of the possible mechanisms of the stimulatory role of hyperuricemia for motivation and directed activity [5]. Nevertheless, there is another side of the coin: the interconnection between UA and fructose metabolism with the increase in the significance of fructose in the formation of energy reserves in the case of high uricemia [9]. In the absence of food, such processes facilitated survival, whereas in modern humans, as overeaters, they become risk factors involved in the development of obesity and metabolic syndrome. Moreover, the latest works of Prof. R.J. Johnson’s research group raise the issue of considering AD “maladaptation of an evolutionary survival pathway mediated by intracerebral fructose and uric acid metabolism” [10]. These authors also point out the necessity for further investigation of the role of UA, the level of which increases during fructose metabolism in AD.

Ambiguous results come from epidemiological and metabolomic studies. Thus, the analysis of nongenetic risks and protective factors, as well as biomarkers for neurological disorders [11, 12], did not allow us to conclude with certainty if there is a need for intensive intervention to influence serum UA levels. Indeed, this work confirmed a lower risk of developing dementia and neurodegenerative diseases (including AD) in individuals with high serum UA. In contrast, the increased risk of developing diabetic peripheral neuropathy and stroke mortality represents a problem. A mixed (protective and risk) effect of UA on neurological disorders was assumed.

The data on the role of serum urate in cognition are summarized in the review of Singh (2018), and the negative influence of serum UA as a risk factor for dementia in the elderly general population is emphasized. Nonetheless, there is a strong need in evaluating this relationship using additional biomarkers of dementia in different cohorts (many of the analysed studies did not include patients with gout and hyperuricemia who received urate-lowering therapies, and there were also significant differences in the age categories), as claimed by the author [13]. The association between elevated serum urate levels and the risks of AD and related dementias, as well as neurodegenerative deaths, was confirmed [14], but Mendelian randomization analysis found no evidence of causality between genetically predicted urate levels and the risk of these neurodegenerative events.

Finally, metabolomic studies [15] showed that serum UA was causally associated with an increased risk of AD. At the same time, in amyotrophic lateral sclerosis, the direction of association was reversed (still, in this case, a false positive result was possibly obtained from the sensitivity analysis). The authors hypothesize that confounding and selection bias explain the controversy in the literature with regard to the role of UA in AD development. Nevertheless, the cited work has been performed at a population-scale metabolome screen not aimed at the biological interpretation of UA effects (including stratification by plasma UA concentration or possible mechanisms and relationships with other markers). The authors do not consider UA to be a general detrimental factor or recommend global hypouricemic interventions but recommend identifying specific individuals or genetic backgrounds that can benefit from urate-lowering interventions [15].

Therefore, there is a need to delineate the beneficial and detrimental effects of UA on the CNS and cognition. In the present work, we aimed to address this issue regarding Alzheimer’s disease by evaluating the positive and negative effects of UA rather than performing a meta-analysis of clinical data concerning UA levels in patients with AD or the significance of UA levels for the risk of AD. These data are abundant, and robust meta-analyses are available [16-19].

At the same time, the conclusions differ regarding the role of UA, and both a protective effect on AD development and a negative impact on its course were detected. Thus, we highlighted the differences between target groups of patients and population subgroups that can cause these differences. Among the mechanisms underlying the neurotropic effects of UA, we described those directly related to AD pathogenesis. Lastly, the data on the molecules that can be considered UA analogues that exert a protective effect in AD and can exhibit advantages in terms of safety (absence of previously mentioned negative cardiovascular and metabolic effects) or additional mechanisms of efficacy not inherent in UA. Since this review was primarily focused on uric acid and its mechanisms, the influence of such molecules on adenosine receptors that are not the target of UA [20] was not taken into consideration.

Notably, UA was included in the shared metabolite signatures among the three diseases associated with neurodegeneration: AD, Parkinson’s disease, and amyotrophic lateral sclerosis [21].

UA is used as one of the markers in clinical trials of drug efficacy in AD [22], and its effects are similar to those of the widely studied drug edaravone [23].

Paganoni and Schwarzschild (2017) proposed the hypothesis that urate may represent a shared pathophysiologic mechanism across neurodegenerative diseases [24]. These findings indicated that antioxidant properties are a mechanism of neuroprotection in several preclinical models. Moreover, an enhanced understanding of the molecular pathways associated with the protective role of UA may lead to the identification of new therapeutic targets for neuroprotection. Given the controversial role of UA in metabolic syndrome and cardiovascular diseases, just this approach seems to be the most reasonable.

## 2 INTERCONNECTION BETWEEN URIC ACID AND ALZHEIMER'S DISEASE IN DIFFERENT TARGET GROUPS OF PATIENTS AND POPULATION SUBGROUPS - POSSIBLE REASONS FOR DIFFERENT URIC ACID ROLE

### 2.1 Gout and Alzheimer's Disease Risk

The previously known phenomenon of a decreased risk of dementia in patients with gout has been confirmed by the results of a recently performed meta-analysis. Since any case of gout is the result of long-term consistent hyperuricemia, this may represent a more reliable interconnection between UA and the brain compared with the general population. Thus, a meta-analysis of six cohort studies involving more than 2,300,000 individuals published between 2015 and 2022 revealed that the risk of all-cause dementia, AD, and vascular dementia is decreased in gout patients (*e.g*., for the risk of AD in gout patients, (RR = 0.70, 95% CI (0.63, 0.79)). Since the quality of evidence was considered to be low and very low, further studies of this interconnection are needed [25]. Heterogeneity was also detected in the data of the meta-analysis described by Wang *et al*. (2023), who confirmed a decrease in the risk of AD not only in patients with gout but also in patients with hyperuricemia (for the risk of AD in such patients, (RR = 0.69, 95% CI (0.64, 0.72)) [17]. Reduced risk of dementia in gout was also seen in Korean patients [26, 27]. The size of these studies was much smaller; nevertheless, stratification was made, allowing for the differentiation of UA effects between patient categories. Specifically, an inverse association between gout and the risk of overall dementia (HR = 0.71, 95% CI (0.52, 0.97)) and AD (HR = 0.67, 95% CI (0.46, 0.97)) was observed in the elderly male group (age ≥65 years) [26]. These data partially align with the analysis [27], indicating that patients with both gout and dementia are more likely to be women. Additionally, these patients had a wide range of comorbidities and more often took gout-related drugs (allopurinol, febuxostat, nonsteroidal anti-inflammatory drugs, and steroids) than patients with gout without dementia. The limitation of these data in the context of our review is that all-cause dementia cases were analysed rather than subdivisions between dementia types.

From the other point of view, not only consistent hyperuricemia but also a chronic proinflammatory state should be considered within the continuum of gout and dementia. Indeed, the incidence of dementia was found to be higher in patients with gout (HR = 1.60 (1.38-1.85)), and the risk was time-varying, peaking in the first 3 years after gout diagnosis and then decreasing [28]. Remarkably, among asymptomatic individuals, there was an inverse association between UA and dementia incidence (HR = 0.85, 95% CI: 0.80-0.89), with no time dependence. An additional advantage of this study is the use of magnetic resonance imaging, which revealed lower brain reserve (smaller global and regional brain volumes) together with markers of higher brain iron (which is also described below) among gout patients. These changes are associated with increased vulnerability to neurodegenerative processes [28]. Gout was independently associated with a 15% greater risk of incident dementia in elderly patients, according to the results of the observational cohort study using a national health insurance database [29]. The authors also link it to the involvement of UA in the cardiovascular continuum, to the detrimental role of xanthine oxidase (XOD) in increasing oxidative stress, or to the intensified inflammatory process (still, there are no data on biochemical markers).

Inflammasomes, particularly the NLRP3 (NLR family pyrin domain containing 3 inflammasomes), are recognized to be involved not only in gout but also in AD pathogenesis, leading to a proinflammatory state with microglial damage. The NLRP3 inflammasome is even considered a potential target for AD counteraction [30].

The data originating from *in vivo* studies showed that constantly increased uricemia (a high-UA diet) triggers the expression of proinflammatory cytokines, activates the toll-like receptor 4 (TLR4)/nuclear factor-kappa B (NF-κB) signaling pathway, and increases gliosis in the hippocampus. NF-κB is considered to be a central link between UA and cognitive dysfunction [31]. Thus, there is a need for further research in this field.

### 2.2 Different Roles of Uric Acid in Alzheimer's Disease and Vascular Dementia

The dependence of UA influence on the etiology of dementia has been described in the review [18], the authors of which emphasize that “serum uric acid may modulate cognitive function differently according to the etiology of dementia.” In contrast to the protective effect of AD, in vascular dementia, high blood UA levels negatively influence the disease course. The interconnection of high UA levels with inflammation can be of special importance in vascular dementia [18].

Thus, the risk for dementia was increased in gout patients, being especially high for vascular dementia (average HR = 2.41, 95% CI: 1.93-3.02, compared to all-cause dementia) [28]. The HR for vascular dementia in gout patients was 2.31 (95% CI 1.02-5.25) in the nonelderly male group in a population-based study [26]. A highly significant increase in vascular or mixed dementia risk (in contrast to AD risk) was detected in community-dwelling older people with high uricemia (described in more detail in section 1.4) [19].

A comparison of plasma UA levels in patients with AD and vascular dementia revealed decreased UA levels in the latter group of patients [32], which was associated with oxidative stress in AD and the consequent depletion of UA, an endogenous antioxidant.

The study of the involvement of oxidative stress in vascular injury in older persons without or with very mild cognitive impairment showed that UA was decreased within the quartiles of patients stratified by carotid intima-media thickness as a vascular aging marker. UA was not independent of the other factors of vascular risk, in contrast to vitamin C plasma levels [33]. These data might be of interest concerning the interconnectedness between urate and ascorbate and the ability of the former to partially replace the latter in the absence of its synthesis [5] (these aspects are also discussed below), as well as the involvement of both substances in vascular pathology.

The interrelationship between UA, lipid metabolism parameters, and dementia was considered in the study [34]. Notably, in these patients, uricemia was substantially and highly significantly reduced only in the subgroup of AD patients, whereas in the patients with vascular dementia (as well as in the entire group of patients with dementia), it did not differ from that in the healthy controls. Moreover, independent of statin use, total blood cholesterol levels were decreased in all subgroups of patients with dementia. Still, high-density lipoproteins (HDL) levels were significantly reduced only in the vascular dementia group but not in the AD group. A negative correlation between plasma UA and HDL was observed but reached a statistically significant level only in the entire group of patients with dementia. The authors noted that, to a small extent, the negative effects of the reduced concentrations of UA on oxidative stress may be compensated for by the beneficial influence of a relative increase in HDL. The molecular mechanisms connected with these phenomena in AD patients were not the aim of the cited study; nevertheless, it revealed a distinction between AD and vascular dementia in the context of changes in uricemia [34].

In order to identify metabolic changes before dementia diagnosis, cognitively healthy participants were followed up for 5-20 years, and cognitive assessments for dementia identification and serum metabolomics analysis were completed every 5 years [35]. Remarkably, UA was among the three serum metabolites that contributed to the separation between demented patients and controls and between incident cases and controls (two other metabolites were 3,4-dihydroxybutanoic acid and docosapentaenoic acid). An increase in serum UA was found in patients with dementia. The majority of them were diagnosed with AD, but there was also a significant proportion of patients with vascular dementia. In contrast to the previously cited studies, differentiating between these two types of dementia was not possible [35], which may be due to the relatively small sample size (in accordance with the aim and type of the study) or the early stages of the disease in the examined patients. The compensatory character of high UA levels in this period can be assumed, but further research is needed.

### 2.3 Alzheimer's Disease and Uric Acid in Males and Females

There are recognized sex-specific differences in the physiological regulation of urate homeostasis [36], and the generally accepted normal serum UA values are between 208 and 428 μmol/L in adult males and postmenopausal women and between 155 and 360 μmol/L in adult females (while there are strong arguments in favor of implementing the recommendable upper threshold value for serum uric acid as < 360 μmol/L for all subjects, given the possible destructive cardiovascular and metabolic effects of high UA levels) [37].

Among genetic factors, significant single nucleotide polymorphisms (SNPs) associated with serum UA concentrations are related to the SLC2A9 gene encoding a transporter involved in renal UA excretion, and the variance in uricemia explained by genotypes differs between females and males [38]. The uricosuric effect of estrogens (verified both through the menstrual cycle and after the intake of synthetic estrogens, which decrease uricemia in most individuals of both sexes) has long been known [39]. Experimentally, estradiol was shown to inhibit XOD [40]. At the same time, testosterone significantly increased plasma UA levels in postmenopausal women, while endogenous testosterone changes due to Leydig cell stimulation produced no definite effect in male subjects [39]. In ontogenesis, a dramatic increase in UA occurs in adolescent boys, and in such a way, sex differences in uricemia are established [41]. Concerning the older age groups, a 25-year follow-up of a Mediterranean population showed a significant increase in serum UA levels with age, and hyperuricemia developed in 1/4 of the recruited individuals. The female participants in this study were all in the postmenopausal period, and increased serum UA over time, as well as the occurrence of new-onset hyperuricemia were independently associated with female sex [42]. A study of Japanese centenarians revealed a positive correlation between uricemia and aging, and this biochemical value progressively increased among the 70-79, 80-89, 90-99, and ≥100 years old age groups [43].

The generally recognized sex dependence of cardiovascular morbidity and mortality is also modulated by serum UA levels. Thus, studies in the Chinese population not only confirmed a higher prevalence of hyperuricemia and hypertension in males but also showed that they (particularly middle-aged individuals) were more susceptible to hypertension. At the same time, this association was not detected in females [44]. On the other hand, higher uricemia was more strongly associated with major cardiovascular adverse events in women than in men with acute coronary syndrome (the interconnection of UA with the increase in cardiovascular risk during the menopausal period is obvious, but the other mechanisms are not sufficiently understood) [45]. A detailed analysis of these issues is beyond the scope of the present review. Nevertheless, they cannot be indifferent to CNS injuries and dementia development. As mentioned above, there is evidence of a greater protective effect of UA on the CNS in male patients with gout: in the study [26], a significant inverse association between gout and the risk of overall dementia was observed only in the male subgroup, while the study [27] showed that patients with both gout and dementia were more likely to be women.

Similar phenomena were also observed in subjects other than patients with gout. Thus, in a prospective cohort study of community-dwelling adults (over ten years of follow-up), it was also shown that higher baseline serum UA in males was associated with attenuated declines in attention and visuospatial abilities and a similar tendency was observed for the decline in language skills (the effect of diuretics and drugs used to treat hyperuricemia appeared not to be significant as well as excluding subjects younger than 50 years). In females, there were no associations between serum UA and cognition or its change over time; nevertheless, the adjustment for dietary factors influenced these associations. This factor appeared to be the consumption of animal protein, and in this case, higher baseline serum UA was associated with poorer performance on measures of visuospatial abilities and language. The same trends were observed in males [46], and these phenomena may reflect interconnections with metabolic syndrome, especially since, in this study, the intake of added sugars and animal protein was associated with higher uricemia, while the intake of dairy products - with significantly lower uricemia.

The same patterns were observed in the sample of healthy middle-aged (younger than 65 years) participants in the ELSA-Brasil cohort study [47] namely the level of serum UA was associated with better performance on an executive function test (Trail Making Test version B) in males but not in females. In this study, participants who were taking hypouricemic drugs or drugs that could influence cognitive performance and those with stroke in anamnesis were excluded. The authors emphasize that the interconnection between UA level and dementia risk may differ according to age and disease duration [47] and the comment for this article [48] indicated the necessity of stratifying the sample by the criterion of hyperuricemia or normouricemia to obtain a clearer result.

In a robust review, there is a stratification of the groups of subjects by the directionality of the connections between uricemia and cognitive function changes; however, this work was not focused on AD and covers the wide spectrum of pathological states and categories of subjects [49].

Increased uricemia was also linked to a reduced risk of cognitive impairment in the male population after a meta-analysis of prospective cohort studies (nine reports including 488,915 participants and 5516 patients with cognitive impairment with a median follow-up of 8.8-22 years) [16]. Blood UA levels appeared not to be related to the risk of cognitive impairment, while the combined RR of cognitive impairment events in the highest uricemia subgroup compared to the lowest quartile was 0.81 (95% CI: 0.70-0.92, *p <* 0.001). There was also a linear negative association between blood UA concentration and cognitive impairment risk in the male subgroup. Interestingly, these data did not support the frequently anticipated curvilinear relationship between blood UA levels and cognitive impairment.

A tendency toward a decrease in UA in brain tissue was also seen only in male patients with AD (these data based on analysis of human postmortem samples are discussed below) [50].

The differences between older males and females in the longitudinal association of baseline uricemia with brain amyloid accumulation were evaluated recently [51]. In amyloid positive patients, uricemia was only inversely correlated with the annual change in amyloid buildup in males. At baseline, there was no relationship between serum UA levels and brain amyloid deposition in the examined subjects of either sex, and there were no sex-related differences in the annual change in amyloid accumulation in subjects without abnormal amyloids. The results were obtained in an observational study; thus, there are no data found on additional biochemical markers or mechanisms explaining the presence of the positive effect only in males [51].

At the same time, there are several reports of the beneficial influence of high blood UA on cognitive function in females. Thus, in the Prospective Population Study of Women in Gothenburg, Sweden [52], with a follow-up of more than 44 years (initial age of participants equal to 38 to 60 years), higher uricemia was associated with a lower risk for late-life dementia regardless of its subtype (for AD and vascular dementia). Adjustments for age, socioeconomic status, education level, body mass index, alcohol consumption, smoking, hypertension status, triglycerides, cholesterol, and estimated glomerular filtration rate (eGFR) were made in this study. Notably, a lower risk was observed only in the subgroup of participants in the highest serum UA category, while in the middle category (with serum UA in the range of 211 to 270 µmol/L), there was no such effect, which the authors explain as the possible insufficient antioxidant effect of such concentrations. The U-shaped association between uricemia and vascular dementia risk is supported by the outcomes of this research (even though the level of significance was not high) and may be interconnected with the incidence of stroke in the sample: the risk for dementia is greater at low UA blood levels after stroke, while high and pathologically increased levels of this metabolite may be related to other cerebrovascular risk factors and a worse prognosis. Since the incidence of hyperuricemia was very low in this study (5.5%), further research is needed [52].

Significant associations were found not only in population-based studies but also in targeted examinations of female patients with dementia [53], with the participation of patients with mild cognitive impairment or AD. The advantage of this approach is the availability of data on AD biomarkers in cerebrospinal fluid (CSF) from the Alzheimer’s Disease Neuroimaging Initiative (ADNI) database. Higher uricemia appeared to exert protective effects on longitudinal cognitive decline, which was partially interrelated with the influence of the abovementioned biomarkers and was more prominent in female patients. Alleviation of the detrimental effect of Aβ_1-42_ and tau by increased levels of UA was shown, and the effect on tau was detected only in female and not in male subjects. The authors distinguish between the significance of sex-related factors for the risk of AD and the risk of vascular cognitive decline [53]. The latter may be increased as uricemia rises, and in male subjects, the prevalence of other vascular risk factors is well known. Decreased performance on tests, the results of which are more frequently changed in vascular pathology, was also observed in females with higher uricemia [54]. Moreover, the direction of high levels of UA action (assessed by beta coefficients) differed between female participants with mild cognitive impairment or AD and baseline healthy females (in which higher uricemia was associated with faster cognitive decline). The authors suggest that the possible vascular pathology is related to these issues [53]. The negative influence of a greater cerebrovascular burden in males and the respective influence of vascular dementia development have also been discussed in other articles [55].

In the case of gout with an intensified inflammatory process, a positive association between gout and incident dementia in elderly individuals was more significant for females [29].

Further clinical studies within the ADNI database have shown that in females with mild cognitive impairment, increased uricemia may interact with apolipoprotein E4 (APOE4) to alleviate longitudinal metabolic changes and cognitive decline. The aim of this study was to estimate brain metabolism using 18F-fluorodeoxyglucose positron emission tomography images (hypometabolic convergence index). Notably, as in the abovementioned study, in this work, higher serum UA levels exhibited a detrimental effect in participants with normal cognition, while a protective effect of high uricemia was observed in females with mild cognitive impairment and was accompanied by a slower decline in cognitive scores, with this effect being present only in APOE4 carriers and partially mediated by the influence on AD-related hypometabolism (measurement of the hypometabolic convergence index). The authors regard the possible antioxidative effect of UA as especially significant at this stage of cognitive disorders. Similarly, the sex-related differences in the effects of UA on males and females reported by Lee *et al*. (2021) are associated with the various levels of vulnerability of males and females to oxidative stress. The decrease in the protective antioxidant effect of estrogens after menopause is well-known, but there are other differences. The authors emphasize the sex-related differences between patients with AD and patients with Parkinson’s disease, who are predominantly female and male, respectively, and that UA has protective effects on Parkinson’s disease; and in contrast, these effects were observed only in males [56]. Sex-related factors may modulate the interplay between pathogenic proteins (Aβ or tau *vs.* α-synuclein) and prooxidants or antioxidants, including UA [55]. Still, the specific molecular mechanisms of these interactions remain to be elucidated.

Assessment of the blood redox status of patients with AD (males and females were included) in accordance with their ApoE genotypes showed that the epsilon4 allele carriers (patients with AD as well as control volunteers) are characterized by lower uricemia and lower catechol-O-methyltransferase activity in erythrocytes [57]. Under such conditions, intensified oxidative deamination of circulating catecholamines by monoamine oxidase-B may be accompanied by an increase in reactive oxygen species. The cited study addressed only the blood redox status [57]; however, the authors indicate that similar processes within the brain are possible in principle, and catecholamines cause an accumulation of reactive oxygen species in hippocampal neurons exposed to Aβ [58]. On the other hand, UA and catecholamines may exert similar and possibly interconnected effects on lipid peroxidation [5, 59]. In the context of the abovementioned phenomena, which could be linked to a decrease in monoamine oxidase activity through a negative feedback mechanism), other mechanisms also exist [60]. Nevertheless, direct evidence of their significance in AD still needs to be obtained.

Analysis of the interconnection between homocysteine metabolism, steroid hormones and the risk of AD development showed that this risk was associated with high serum concentrations of UA and homocysteine (which positively interact in this context) and low concentrations of estradiol (in patients with AD and healthy control subjects) [61].

In general, direct neuroprotective effects of estrogens have been demonstrated. For instance, 17β-estradiol did not change Aβ secretion or pTau accumulation *in vitro* but promoted neuronal plasticity and alleviated the astrogliosis-like phenotype. Such effects are thought to be the reason for the ambiguous results concerning this estrogen efficacy in clinical trials [62]. Such fundamental targets as astrocytic Cx43 gap junctions have been shown to mediate the cytoprotective effects of estrogens in the brain [63]. Nevertheless, data on the interrelationship or modulation of these effects by UA are practically absent in the available literature.

Another aspect of high importance is the involvement of XOD in the generation of ROS and consequent prooxidant processes. The interplay between estrogens or androgens and UA at the level of the endothelium is described in the review [64]. Only in the endothelium does xanthine oxidase (XOD) activity lead to the concomitant generation of free radicals [65], while estrogens can promote NO release and prevent the formation of free radicals. The other mechanism of endothelial protection may involve a decrease in the UA level in the bloodstream, which was partially discussed above. Androgens may exert both vasoprotective and vasoinjurious effects [66] and cause secondary effects after the conversion to estrogens; thus, andropause and menopause increase uricemia and endothelial damage [64], but there is no discussion of the interconnection of these processes with UA effects within the brain.

### 2.4 Cardiovascular Risks, Kidney Function, Serum Uric Acid, and Dementia

It is not expedient to characterize dementia risks without the context of cerebrovascular burden. Thus, diastolic blood pressure (BP) was consistently correlated with blood directly accessible Aβ40 levels in patients with AD, subjects with mild cognitive impairment and healthy controls (cross-sectional study [67]). The levels of Aβ_40_ (both directly accessible and recovered from the plasma matrix) were also strongly associated with creatinine levels and, to a lesser extent, with UA, urea, and homocysteine levels. Since there was a significant correlation between creatinine and these parameters, the latter variables were not included in the regression models. Nevertheless, the authors emphasize that the influence of the state of the excretory renal function is not the only factor that should be investigated, and the creatine/creatinine energy cycle in the brain and muscle could have an interrelationship with amyloid physiology [67]. In addition, from our point of view, the primary negative influence of high uricemia on the kidney and vessels with a possible increase in BP [6, 8] should be considered in this context.

Indicatively, when the impact of renal function was considered in the assessment of the correlation between uricemia and cognitive impairment (serum UA to serum creatinine ratio, SUA/SCR), it was shown that individuals with lower SUA/SCR ratios had a higher risk of cognitive impairment (the analysed sample included older adults aged 60 and above in the United States from the National Health and Nutrition Examination Survey database). The examined subjects from the fourth quartile of SUA/SCR (after adjusting for covariates) had a 45% reduced risk of cognitive impairment [68].

Stroke risks and their modulation are additional important factors within the continuum of UA exchange and dementia development. An assessment of the longitudinal association between serum UA levels and incident dementia in a large cohort of healthy community-dwelling older people confirmed an increase in such risk (especially for vascular or mixed dementia, while it was not significant for AD) in patients with high uricemia. This association was supposed to be affected by interim strokes [19].

Among the components of standard antihypertensive therapy, thiazide diuretics are commonly characterized as drugs that can cause hyperuricemia as a side effect. Notably, a meta-analysis [69] confirmed the beneficial influence of diuretics on cognitive health and for thiazides that could be linked specifically to hyperuricemia (while potassium-sparing diuretics possess a different mechanism that partially consists of maintaining kalemia with a possible favorable influence on amyloid formation, and in such a way, two subgroups of diuretics with opposite effects on blood potassium levels exert a beneficial effect on cognitive health during aging). In general, an inverse association between the use of thiazide and potassium-sparing diuretics (alone or in combination) and the incidence rate of dementia was established in the study [69].

The cardiovascular risks of high fructose consumption are generally accepted, and such consumption has also been linked to high uricemia. The involvement of XOD in these processes was described [70]. The enzyme, *via* increased generation of reactive oxygen species and decreased NAPDH concentration, can contribute to the reduction of NO synthesis. Consequently, cerebrovascular endothelial dysfunction is exacerbated; while excessive, NO synthetized by overactivated nNOS causes neurotoxicity. A greater risk of all-cause dementia and AD dementia was confirmed in subjects with high fructose consumption in this study.

### 2.5 Smoking, Serum Uric Acid, and Dementia Risks

The benefits of tobacco smoking cessation are unquestionable; however, the relationship between neurological disorder risk and smoking is an example of a multicomponent and ambiguous continuum [11, 12], and that is also true for the relationship between smoking and gout development. A consensus has not been reached as to the effect of smoking on the serum UA concentration and the risk of gout; nevertheless, there are numerous reports concerning decreased uricemia in smokers [71]. The increase in hyperuricemia risk was clearly shown after electronic cigarette exposure [72]), and smoking is associated with an elevated risk of multiple sclerosis and dementia. At the same time, smoking is simultaneously associated with a reduced risk of Parkinson's disease and decreased uricemia [11, 12, 71]. This finding illustrates the diverse effects of UA on different types of brain dysfunction.

### 2.6 Education Level, Serum Uric Acid, and Alzheimer's Disease

The relationships among educational level, serum UA and AD are extremely interesting from several perspectives. First, the protective influence of high educational attainment against age-related cognitive decline and, to a lesser extent, against AD is known [73]. Second, the influence of UA on intellectual activity is mostly realized through increases in motivation, activity intensity, professional productivity, *etc*. (while it is virtually impossible to change intellectual abilities *per se* due to the influence of a single metabolite) [2] that were also previously addressed in our review [5]. Thus, the following question logically arises: could the primary influence of uricemia on the subject’s motivation to obtain education contribute to the decrease in dementia risk later in life?

There are data that partially confirm this interrelationship. Thus, educational level influenced the relationship between UA and the risk of dementia according to the results of meta-analyses [74]. Notably, in contrast to academic level, age, body mass index, diabetes mellitus status, hypertension status, and smoking status did not influence the identified risk estimates. A significant association of uricemia with AD (but not with vascular dementia) was confirmed in this study, and different gradients of concentrations of UA quantitatively assessed the risk of dementia. The prevalence of dementia increased with lower educational attainment.

As a result of two-sample Mendelian randomization, both genetically predicted higher years of schooling and genetically predicted higher urate levels appeared to be factors suggestively associated with the risk of AD (increased years of schooling were associated with a reduction in risk, while increased urate levels were associated with a slight increase in risk) [75]. Nevertheless, the direct interrelationships between uricemia, educational level, and AD were not assessed in this study. The authors also emphasize that an association between UA and AD still remains elusive and needs to be investigated further.

### 2.7 Low-grade Inflammation, Serum Uric Acid, and Dementia Risks

Since it is unreasonable to discuss many nosological states, including dementia, outside the context of low-grade inflammation, of special interest is the work of Jiang and co-authors (2023), who have shown that the cognitive function benefits of increased uricemia are present only under low-grade inflammation conditions but not under high-grade inflammation conditions (assessed by the criterion of high-sensitivity C-reactive protein). Nonhyperuricemic adults (mean age 58 years, both sexes) participated in the study. Among those with low-grade inflammation, higher UA concentration was associated with better cognitive function after 4 years (hypertension and stroke history were used as covariates) [76].

In elderly individuals with mild cognitive impairment (≥ 60 years), a relationship between lower serum UA levels and a greater risk of mild cognitive impairment was established. In contrast, this relationship was not observed in a subgroup of participants with obesity. In this subgroup, higher uricemia and hypersensitive C reactive protein levels were detected, and there was a statistically significant positive correlation between these values. Thus, the authors concluded that obesity-induced inflammation may weaken the relationship between lower uricemia and the risk of mild cognitive impairment. The direct mechanisms were not assessed in the study; however, adjustments were made for vascular risk factors, and there was no linear correlation between these factors and uricemia. Adipocytokines, which are increased in patients with mild cognitive impairment and AD, are thought to be possible mediators of this relationship, but they have not been directly assessed [77].

There are other examples of the influence of the proinflammatory milieu on the neurotropic effects of UA. For instance, the direction of the association between serum UA and depressive symptoms was the opposite in representatives of the general population with and without low-grade inflammation [78]. However, these aspects are beyond the scope of the present review.

In general, the data analysed above are of high clinical significance; nevertheless, there is a lack of information about certain molecular mechanisms linking UA metabolism and low-grade inflammation in the context of brain function and dementia development.

### 2.8 Malnutrition, Uricemia Changes, and Alzheimer's Disease

Apart from the abovementioned relationships, it is logical that decreased uricemia can develop in a rapidly progressive course as a consequence and marker of malnutrition [18, 79]. This is especially important in the case of dementia, which is a generally accepted risk factor for malnutrition, and in the case of aging itself, in the context of so-called “anorexia of aging.” Vicious circles can be formed since malnutrition may accelerate cognitive impairment, and in this situation, UA is not considered as active mediator but rather a metabolic marker [18].

From the other point of view, XOD inhibition is associated with intensified reutilization of available hypoxanthine through the purine salvage pathway and its use to resynthesize adenine nucleotides, including ATP. Notably, in patients with cardiovascular diseases, withdrawal of XOD inhibitors leads to an extreme increase in cardiovascular events, and the reason for this phenomenon is just the rapid removal of increased ATP by discontinuation of treatment [80]. Based on these properties, the benefits of XOD inhibitors in treating aging-related malnutrition could be investigated. Allopurinol is considered promising for improving functional gains and reducing sarcopenia; nevertheless, the risk of frailty was not decreased by this drug in older adults with gout, whereas a reduced risk of persistent physical disability was shown [81].

## 3 URIC ACID IN DIFFERENT TISSUES AND BIOLOGICAL FLUIDS IN ALZHEIMER'S DISEASE

### 3.1 Uric Acid in Brain Tissue

Modern methods allow unprecedented approaches for evaluating the levels of brain metabolites, including UA. This is vital for the substantiation of any pharmacological effect and could be crucial for the investigation of UA's diverse and occasionally controversial role.

#### 3.1.1 Postmortem Data Concerning Uric Acid in the Brain

There is evidence of UA changes in the brain obtained from human postmortem samples. Thus, alterations in purine metabolism were registered at the early stages of AD, being independent of neurofibrillary tangles and Aβ plaques [82]. These alterations were stage-dependent and region-dependent. For instance, in the frontal cortex, there were significant reductions in adenosine, guanosine, hypoxanthine and xanthine concentrations (while in parietal and temporal cortices, an opposing pattern was registered). The activity of 5'-nucleotidase, adenosine deaminase and purine nucleoside phosphorylase was also pathologically changed, still there is no data about UA concentration [82]. Such data are present in the article [50], which confirmed the differences between the subtypes of dementia in regard to purine metabolism: the inverse association between urate levels and Parkinson's disease, and, to a lesser extent, AD, while increased urate levels in the brain were registered in patients with dementia with Lewy bodies. Notably, sex-specific differences in UA involvement were also observed within this study: only in males with AD did UA levels in cortical and striatal tissue trend lower than those in controls (while among the control subjects, increased urate in males *versus* females was observed). Urate precursors were also assessed in these samples, and relative increases in inosine and adenosine were detected in males [50]. The data from the study provide a basis for the protective role of UA as a peroxynitrite scavenger under conditions of nitric oxide synthase hyperactivation [83]. HPLC with electrochemical array detection revealed a decrease in UA levels in four regions of the human brain that are differentially affected in AD as well as in ventricular cerebrospinal fluid. Moreover, 3,3'-dityrosine and 3-nitrotyrosine were significantly increased in the hippocampus, neocortical regions, and ventricular cerebrospinal fluid.

#### 3.1.2 Experimental Data Concerning Uric Acid in the Brain

In the triple-knockout 3×Tg mouse model of AD, simultaneous direct determination of metabolites using matrix-assisted laser desorption/ionization mass spectrometry imaging revealed a shift toward an increase in hypoxanthine/xanthine/uric acid together with a decrease in AMP and adenine. Notably, a decrease in ascorbic acid was also observed [84]. The interconnectedness between the role of urate and ascorbate is generally recognized (and the interrelation between the loss of L-gluconolactone oxidase and uricase during the evolution of humans when urate partially replaces ascorbate is known) [85]. In patients with AD, a positive correlation was established between the levels of UA and ascorbic acid in the cerebrospinal fluid but not in blood plasma [86].

In the study of glutamate-induced metabolic syndrome development, the increase in liver UA resulted in the increment of UA in the hypothalamus, confirming the availability of the synthesized UA for the brain (although UA under these conditions has a negative role in metabolic syndrome development) [87].

The enhanced linkage of UA from the gut to the brain was considered a mechanism of the protective effect of the probiotic intervention on the model of neurodegenerative disorders induced in mice by a high-Fe diet. The authors report a significant increase in UA levels in the hippocampus and in the cortical tissue of the treated mice, which also demonstrated reduced AD-associated tau levels within the brain as well as improved spatial learning and memory. Moreover, the beneficial effects of UA, as an important molecule in AD pathogenesis, were investigated. In *Caenorhabditis elegans* overexpressing human p-tau, successful intervention by probiotic enhanced UA transport by upregulating the transporter *wht8* and decreasing the activity of *oat-1* (a homolog of OAT1) that is important for UA excretion. Remarkably, UA exerted a lifespan-extending effect on these nematodes [88]. Other studies have confirmed the beneficial effects of UA on different AD models, but direct measurements of UA in the brain are not always available (for instance, [89]).

Additionally, it should be noted that there are data concerning the negative effects of UA *in vivo*. Thus, studies on models of hyperuricemia in rats and intracerebroventricular UA administration in mice verified that UA uptake by hippocampal mitochondria further disrupted their bioenergetic function and decreased ATP production and cytochrome c oxidase activity [90].

The other aspect of UA kinetics is its concentration in the cerebrospinal fluid (CSF), and the availability of such samples and the possibility of dynamic observations necessitate a larger amount of such data. The relationship between blood and CSF concentrations and the possible influence of UA on BBB permeability are also of great interest.

### 3.2 Uric Acid Influence on BBB, its Presence in Cerebrospinal Fluid and Problems of Transport to the Brain

The ratio of UA concentrations in the blood serum and CSF is 10: 1 [91]. For 99% of the examined subjects, the dependence y = 0.1x ± 0.010 mm/L holds for, and x and y are the UA in the blood and CSF, respectively [92]. A similar relationship was shown for patients with AD [86], which substantiated the concept that CSF UA is determined by plasma UA and BBB integrity. CSF UA and CSF ascorbic acid were positively correlated (and such correlation was not observed in plasma).

As is well known, hypoxanthine is an especially significant metabolite of the purine pathway within the brain, where hypoxanthine phosphoribosyl transferase is highly active and severe disorders arise in its deficiency (Lesh-Nyhan syndrome); thus, hypoxanthine salvage and transport are of great importance.

The opposite transport directions of xanthine and UA were observed in a clinical study addressing ventricular-lumbal gradients for certain metabolites. A decrease in urate concentration was found in successive CSF specimens (cisternal CSF and lumbar CSF; some of the samples were obtained from patients with dementia), while there was an increase in the concentrations of hypoxanthine, xanthine, and creatinine [93].

The previously obtained data summarized in the review of Tovchiga and Shtrygol' (2014), is mostly evidence that there is no active transport of UA from the blood to the brain in most cases [5]. Recent publications also partially support this fact [94]. Nevertheless, it has recently been demonstrated that UA can be transported from the blood to the CSF *via* choroid plexus epithelial cells and then from the CSF to the brain parenchyma *via* ependymal cells [95]. Transporters URAT1, GLUT9, and BCRP are expressed in the choroid plexus epithelial cells and/or ependymal cells of human and mouse brains [96-98]. At the same time, BCRP/ATP-binding cassette transporter G2 (ABCG2) is considered to be the main urate transporter at the BBB. It is expressed on the luminal membrane of capillary endothelial cells and is believed to excrete brain urate into the blood [97, 99].

The experimental studies cited in section 2.1.2 indirectly confirmed the possibility of transporting UA from the blood to the brain by describing the increase in UA in the brains of hyperuricemic mice.

As summarized in the review of Tovchiga and Shtrygol' (2014), [5], there is evidence of the presence of XOD in the brain; thus, the formation of uric acid *in situ* is possible, and XOD can be measured in brain tissue [100]. However, the activity of this enzyme in brain capillaries is much greater than that in brain tissue [5, 100]. Microvascular endothelial cells are especially rich in oxidoreductases [101]. Thus, UA synthetized locally in the brain vascular network can also be considered in the context of its availability for the brain.

In addition, xanthine oxidoreductase is located not only in the cytoplasm of the cell but also on the outer surface of the endothelial cell plasma membrane, and this enzyme is highly important during oxidative stress. Moreover, during reperfusion after ischemia, XOD can be released into the systemic circulation from the liver and intestine, where it can bind to the plasma membrane of endothelial cells *via* surface glycosaminoglycans, exerting a negative effect on distant organs [101]. These processes may mediate vessel injury and detrimental effects on different systems, and this may reflect the negative role of XOD rather than the involvement of high concentrations of UA per se [5].

There is also evidence of CSF UA concentrations in AD patients. Thus, a significant decrease in this value was observed in patients with Alzheimer-type dementia, in contrast to patients with other dementia subtypes, and the ratio of the concentrations of UA to xanthine in the CSF correlated significantly with the ratio of the concentrations of UA in the CSF and blood serum. These changes are considered markers of impaired brain metabolism, in contrast to the changes in patients with vascular dementia of the Binswanger type, in which the increase in CSF UA level was attributed to an impairment of the BBB [102].

In contrast to these data, the study of Degrell and Niklasson (1988) showed a significant elevation of UA in the CSF of patients who have senile dementia of the Alzheimer's type, whereas this did not occur in patients with presenile dementia. Notably, xanthine levels were increased significantly in both groups, while there was no difference in hypoxanthine concentration. Creatinine concentrations were also elevated in both groups [103]. The latter may represent an indicator of metabolic shifts since, as described in section 1.4, the creatine/creatinine energy cycle in the brain and muscle may be linked to amyloid metabolism.

Discussions and feedback on the interplay between UA and the BBB began in the 2010s [86, 104] and are currently ongoing [105, 106]. The controversial issues raised earlier [104] are still actively discussed, for example, whether the increased UA concentrations within the CNS reflect the primary disruption of the BBB or how high blood UA levels specifically influence on the integrity of the BBB in different CNS disorders, including counteraction to pathological shifts in BBB permeability as well as to proinflammatory processes. In some cases, maintenance of the protective role of the BBB may be more important for neuroprotection than CNS levels of urate [104], and UA is known to prevent endothelial cell adhesion and the penetration of inflammatory cells in the CNS [5, 107]. Moreover, the influence of UA on BBB permeability can lead to both positive and negative results depending on the specific pathological process. Thus, the opposite results were observed in models of ischemic stroke [108] and hemorrhagic shock [109].

In the b.End3 brain endothelial cell line exposed to peroxynitrite, UA did not influence the TNF-α-mediated increase in the adhesion molecules on the cells but reduced the adhesion of encephalitogenically primed T lymphocytes to these cells [110].

Recent discussions strongly emphasize the necessity of characterizing the implications of systemically circulating UA levels and its production in the brain, as well as the role of the BBB in urate transport under certain pathological conditions. This can allow the determination of optimal uricemia for neurodegenerative disease prevention or management. Even though a strong correlation between UA concentrations in blood and the CVF is predominant, the value of CSF UA measurement has been noted, especially in the context of the possibility of specifying its association with different markers of Aβ and tau metabolism. The possibility of ascertaining the direct or indirect role of the BBB in urate transport and neuroprotection also appears [105, 106].

The other crucial factor is the compartmentalization of uric acid and its transport within the CNS. Just this factor could determine the central effect of UA, and in this case, the role of the systemic pool and uricemia could not be essential. Thus, using uricemia as a main indicator could be the reason for the inability of clinical studies to confirm the associations between UA and cognition.

The extremely important data in this context are given in the article [111]. This work covers the role of ITM2B, the protein that interacts with GLUT9 (the SLC2A9 gene, encoding this urate transporter, has the largest single-gene effect on uricemia), as a molecular link between urate homeostasis and neurodegenerative disorders. ITM2B has been shown to be a regulator of GLUT9-mediated urate transport, with potential roles in both systemic and cellular uric acid homeostasis. Notably, ITM2B is ubiquitously expressed and abundant not only in the kidney and liver but also in the brain, and one of the findings of this study is that the ITM2B-GLUT9 interaction might not affect circulating UA but might influence its compartmentalization within the CNS and other systems. ITM2B variants are linked to familial Danish dementia and retinal dystrophy. Still, these transport systems have a complicated influence on urate transport, and their role in CNS urate homeostasis has yet to be explored [111].

*In vitro* data reported by Cipriani *et al*. (2012) [112] showed that the expression of URAT1 and GLUT9 in mouse astrocytes is crucial for the UA protective effect on the dopaminergic neurons in cellular oxidative stress. At the same time, UA was not protective in pure dopaminergic cell cultures.

Thus, the concepts described above may explain why some studies that addressed genetically determined variations in circulating urate failed to establish a link between these variations and the risk of dementia, for example, the study [113], in which two-sample Mendelian randomization analyses have not confirmed a decrease in AD risk at higher levels of exposure to circulating antioxidants, including UA; or the work [114] that used genome-wide significant single nucleotide polymorphisms for UA as instrumental variables and showed an increased risks (albeit not reaching the level of statistical significance) of AD in lifelong genetically increased uricemia. However, the causal effect of genetically determined serum urate was not confirmed by a Mendelian randomization study [115] on cardiovascular outcomes (as well as on neurovascular outcomes). The aforementioned variations in UA compartmentalization and its availability for the target cells are in addition to the possibility of pleiotropic effects [115]. In case of insufficient supply to the target cells, only the effects toward other tissues would be evident, *e.g*., the negative impact on the endothelium, leading to detrimental cardiovascular and neurovascular outcomes.

### 3.3 Other Aspects of Uric Acid Presence in Tissues and Biological Fluids

#### 3.3.1 Uric Acid Blood Concentration Range in the Context of Alzheimer's Disease

The novel disease concept of “dysuricemia” is proposed in work [3], emphasizing that “uric acid is much more than a metabolic waste product” and that both high and low UA blood levels lead to pathological consequences.

A recent retrospective study showed a progressive decrease in uricemia in a comparison between healthy controls, patients with mild cognitive impairment, and patients with late-onset AD (adjusted for age, sex, body mass index, and serum creatinine levels), which led to a conclusion about the protective role of UA against AD in older individuals [116].

In the context of tauopathies, low concentrations of serum UA were detected in patients with different tauopathies, including AD, and, remarkably, UA serum concentrations and the risk of tauopathy were inversely related [117]. Thus, a low level of blood UA is considered a common risk factor for tauopathies, and a cutoff value of 259 µM was used to distinguish between control subjects and patients with tauopathies. It is supposed to be useful for the initial assessment of tauopathy risk, although its specificity and sensitivity are not high [33].

A negative correlation between UA or glucose and the ratio of plasma Aβ_1-42_/Aβ_1-40_ was established in metabolomic studies of blood plasma from presymptomatic PSEN1-H163Y mutation carriers (hereditary AD) [118].

Upstream purine metabolites, namely, the hypoxanthine concentration in blood plasma, also displayed a decreased hazard ratio with dementia risk, which was regarded as one of the arguments for the neuroprotective effect of UA [119].

#### 3.3.2 Changes in Uric Acid Renal Excretion in the Context of Alzheimer's Disease

A system of urate reabsorption has been formed in kidneys throughout evolution, and despite the high energy cost, more than 90% of the filtered UA is reabsorbed. This is implicated in the maintenance of high levels of uricemia in humans, underlining the biological importance of UA [5, 120].

Renal hypouricemia caused by genetic defects in UA transporters and excessive UA excretion is considered a significant clinical problem, and clinical practice guidelines have been published on this issue [121]. In addition to the generally known consequences of this state, such as nephrolithiasis and exercise-induced acute renal failure (related to oxidant stress from oxygen-free radicals causing renal vasoconstriction, ischemia, and subsequent reperfusion injury), posterior reversible encephalopathy syndrome has been described in such patients and it has been linked to vasogenic edema [18, 122].

The processes described above reflect the diverse biological activities of UA; nevertheless, specific changes in renal UA handling in AD patients have also been reported. Thus, the serum UA concentration was lower, and the fractional excretion of urate was higher in patients with AD than in those with multi-infarct dementia (and also compared with the nondemented control subjects). Uncommonly, fractional excretion of sodium was increased in patients with AD, which was not the result of the drugs taken, comorbidities, or high sodium intake. By the methods available at the time of the study, an increase in the level of natriuretic factors was proven. The fractional excretion of lithium also increased and correlated inversely with mini-mental status examination scores. This biochemical value was even proposed as a diagnostic marker, allowing the authors to determine the severity and, possibly, the rate of progression of dementia in AD and to differentiate AD from multi-infarct dementia [123]. Unfortunately, there is a lack of recently obtained data in this field despite the new possibilities in renal physiology and pathophysiology. At the same time, the involvement of increased sodium chloride intake is supposed to be involved in the pathogenesis of dementia (including AD), being not limited to the risk factors for stroke. The interrelationship between high salt intake and dementia is thought to be realized through the impairment of nitric oxide bioactivity in the cerebral microvasculature [124]. The latter is an important target of UA, and the specific role of UA in the cerebral vascular bed constantly exposed to excess sodium and affected by the corresponding neurohumoral shifts has yet to be determined.

#### 3.3.3 Salivary Uric Acid and Alzheimer's Disease

There has been considerable interest in the use of saliva for diagnostics and as one of the matrices for metabolomic studies. The ability of UA to be measured in saliva has shown great potential, and such measurements are thought to be essential for a number of pathological processes, including neurological conditions. In addition to blood, a reduced UA concentration in saliva is regarded as a marker for AD and impaired cognition [125].

## 4 URIC ACID AND ALZHEIMER'S DISEASE-RELATED GLUCOSE HYPOMETABOLISM

As mentioned above, UA is among the shared metabolite signatures of neurodegenerative diseases, including AD, and purine metabolism shifts are associated with inadequate glucose supply and energy crisis in neurodegeneration (although there is no description of the certain connection points between these processes) [21]. This association is not directly connected with such protective factor for AD as thiamine diphosphate since there was no correlation of this coenzyme with uricemia in patients with AD (there was also no correlation with fasting glucose) [126].

Under the metabolomic approach, UA is considered together with glucose within the pathway of metabolites “Glucose metabolism disorders,” and this pathway demonstrated differences between the presymptomatic PSEN1-H163Y mutation carriers (hereditary AD) and control subjects [118].

Metabolomic studies of the blood plasma of the abovementioned mutation carriers revealed a negative correlation between the ratio of plasma Aβ_1-42_/Aβ_1-40_ in blood plasma and either UA or glucose. Among the annotated metabolites were both hexoses and purine nucleosides (confirmed in two different datasets) [118].

The normalizing influence on AD-related hypometabolism is considered one of the links between the antioxidant effect of increased UA levels and an improvement in cognitive processes in females with mild cognitive impairment (also discussed above), which is specific for sex and stage of cognitive impairment [55].

The similarities of AD, Parkinson’s disease, and Huntington’s disease in relation to disturbances in glucose and purine pathways were further confirmed in a systematic review [127], which also revealed decreased UA in blood samples of patients with AD. Summary of the biochemical pathway changes within brain tissue demonstrated the increased glucose levels with unchanged UA concentrations and unchanged or reduced hypoxanthine and xanthine levels (data summarization is complicated by differences in the studied regions of the brain and methods used in various sources). Increased iron and decreased copper were also confirmed as common features of brain changes in AD [127].

An association between low uricemia and AD-related cerebral hypometabolism was also confirmed by the principally new methods in lifetime diagnostics (namely, multimodal brain imaging) [128]. This study was carried out in nondemented older adults. Moreover, there were no associations between uricemia and Aβ deposition, tau deposition, or white matter hyperintensity volume. The authors also indicate that the causality of the mentioned relationship remains to be confirmed.

## 5 XANTHINE OXIDASE (XOD), URIC ACID, AND RISK OF COGNITIVE IMPAIRMENT

As mentioned above, XOD is a generally recognized source of free radicals generation [65]. Thus, its inhibition is considered to be more expedient than other mechanisms of hypouricemic action due to the additional reduction of oxidative stress [5]. Currently, the benefits of XOD inhibitors in cardiology and nephrology are well known. Importantly, XOD inhibitor use is not always associated with the risk of cognitive decline that could be expected proceeding from uricemia decrease. This was demonstrated during the 5-year follow-up of community-dwelling older adults (≥ 70 years old) treated with allopurinol or febuxostat. In these patients, there were no differences in the change in the composite cognitive score (adjusted for other risk factors for cognitive impairment) [129].

Interestingly, a retrospective cohort study using data from the state insurance system in Korea confirmed not only a decrease in dementia risk in patients with gout but also a reduction in such risk in elderly patients taking febuxostat (whereas no association was detected between dementia and allopurinol use) [130]. Further studies encouraged by this study showed that, for instance, in Taiwanese patients, there was no significant association between AD and allopurinol use (data from a case‒control study). The authors highlight the absence of direct extrapolation of the experimental data showing the ability of XOD inhibitors to protect against oxidative stress in the clinic and the need for further research in this field [131]. A comparison of the influence of allopurinol and febuxostat on the risk of dementia in older adults (a retrospective cohort study) revealed a dose-related reduction in this risk for both drugs [132].

Furthermore, xanthine dehydrogenase/oxidase appeared to be the most efficient target for neurodegenerative disease prevention according to the results of the comparison of the different biological targets [133] (the study was not directly focused on UA exchange). The data on the prescribed drugs from the national health insurance database were analysed after categorization according to their biological targets. An inverse association with the incidence of neurodegenerative diseases (including AD) was observed for XOD inhibitors only, and allopurinol accounted for 23% of the reduction in the incidence of neurodegenerative disease in the fifth year of follow-up. These results substantiate the choice of XOD inhibition as a possible new direction for neuroprotection (drugs repurposing or developing) [133]. Notably, carvedilol, which possesses an additional pharmacodynamic ability to protect endothelial cells from damage initiated by XOD [134], also decreased the risk of neurodegenerative diseases [133]. The similar effect of colchicine (a tubulin alpha/beta chain blocker used in gout treatment) was not confirmed in further analyses, and its effect was considered to be confounded by co-treatment with allopurinol [133]. In addition, certain compounds of herbal origin (including flavonoids) have been proposed as XOD inhibitors with potential applications in mental disorders [135].

Regarding the influence of allopurinol on the CNS, it is notable that continued use of allopurinol was associated with a decreased rate of incident depression (based on data from Danish nationwide population-based registries) [136]. In contrast, gout itself may be independently associated with a greater risk of depression [137].

On the other hand, decreasing uricemia can contribute to a decrease in pathological behaviours, which can be partially mediated by high UA levels. In this context, the possible efficacy of allopurinol in treating neuropsychiatric disorders, including aggressive behaviour and pathological activity, was investigated, for instance, as an adjuvant drug for bipolar disorder treatment (although some studies did not demonstrate sufficient activity) [138]. Allopurinol also appears to be effective in patients with dementia associated with prominent aggressive behaviour not responding to standard treatment. In a case-series study, it successfully eliminated signs of aggressive behaviour (in five of the six patients included in the study) and did not cause any side effects. Either changes in purine metabolites or a decrease in oxidative stress are supposedly the mechanisms underlying this effect [139].

The other important aspects of allopurinol pharmacodynamics were established experimentally. Thus, in a mouse model of brain ischemia, pretreatment with allopurinol reduced microglial infiltration, astrocyte proliferation and nitrosative stress, and a decrease in infarct volume was observed. This is a relevant issue since microinfarctions are common in patients with cognitive decline and dementia. The elimination of oxidative stress within the vascular bed is supposed to be significant in this process [140]. Among the molecular mechanisms of Aβ accumulation, attracts attention the fact that xanthine/XOD free radical generation *in vitro* is related to reduced activity of cathepsin B with a further increase in large amyloid oligomers [141].

Moreover, XOD-derived free radicals inactivate the protease nexin-1 (PN-1) [142], an endogenous thrombin inhibitor and neurite outgrowth promoter, which is considered to exert a protective effect against AD by regulating the sonic hedgehog signaling pathway [143].

Such phenomena may illustrate that the effects of high UA levels in biological fluids and the consequences of high XOD activity can be fundamentally divergent. This was previously known because of the emphasis on the negative role of XOD in endothelia [5]. At the same time, these mechanisms are only a part of a multi-component AD pathogenesis, and XOD inhibitors are not effective under all conditions. Thus, allopurinol and oxypurinol did not attenuate Aβ neurotoxicity in rat hippocampal neurons *in vitro* [144].

Molybdenum deficiency has been revealed in patients with AD due to the dysregulation of copper metabolism (as a biological antagonist of molybdenum). In certain cases, the accumulation of copper not bound to ceruloplasmin can disrupt the transport of molybdenum outside blood vessels, causing mild molybdenum deficiency. Since molybdenum is a cofactor of XOD, it may lead to the decreased activity of this enzyme being the reason for low uricemia in such patients [145]. The other aspect related to such shifts in microelement transport is the pro-oxidant effect of UA, which is considered to be specific for copper-induced LDL oxidation [146]. These phenomena illustrate once more an ambiguous role of UA.

## 6 URIC ACID EXCHANGE AND THE CHOLINERGIC SYSTEM

The interconnection of UA-mediated effects with cholinergic mechanisms seems to be improbable; nevertheless, there is evidence of its existence. Based on computational analysis, Mazumder *et al*. reported that UA may act as a potent inhibitor of acetylcholinesterase (AChE), with a binding affinity higher than that of known inhibitors of the enzyme; the same effect is inherent to adenine, hypoxanthine, xanthine, and 2,8-dihydroxyadenine. The authors associate depleted AChE activity with dementia and cognitive impairment [147]. Nevertheless, these data were not confirmed by direct activity measurements. It is also difficult to assume cholinergic effects in patients with severe hyperuricemia and gout.

Alternately, hypoxanthine (studied in the context of its accumulation in Lesch-Nyhan disease with severe changes in the CNS) was able to influence AChE activity *ex vivo*. Namely, it enhanced AChE activity in the hippocampus and striatum (but not in the cerebral cortex) of young rats but did not change AChE activity in older rats. The serum butyrylcholinesterase activity was inhibited only at the highest concentration in older rats. The authors suppose that the mechanism is linked to the production of free radicals and UA (antioxidants and allopurinol prevented the changes in AChE activity); however, in this work, there are no data about UA concentrations or other direct measurements of its influence on the enzyme [148]. *In vivo* hypoxanthine after intrastriatal administration had a multidirectional and time-dependent influence on AChE activity in the striatum and cerebral cortex but did not change AChE activity in the hippocampus. The direct mechanisms of the interaction with the enzyme remain unclear [149].

On the other hand, data previously obtained *in vivo* have shown that cholinergic agents are able to induce hyperuricemia. In the uricase-inhibited rat, pretreatment with acetylcholine and carbachol, and especially the former after pretreatment with physostigmine, increased the hyperuricemic response, and either M-cholinoblocker or the XOD blocker blocked this increase. Further analysis has shown that cholinergic agents stimulate UA synthesis in organs and tissues other than the liver and gastrointestinal viscera [150]. This is a challenging issue because of the possible modulation of the effects of cholinotropic agents in patients with hyperuricemia, but direct clinical evidence about it is not available.

The targeting of the cholinergic system with adenosinergic and purinergic compounds was successfully realized in the search for multitargeted drugs. New synthetic caffeine analogues were proposed as modulators of the cholinergic system [151].

Given the antioxidant and neuroprotective potential of UA, it is not surprising that it can exert a protective effect on cholinergic transmission. Generally, the attenuation of synaptic dysfunction may be a possible mechanism underlying the beneficial effects of UA [53], and oxidative stress markers are increased and more localized to synapses in the brains of AD patients [152].

In cholinergic synaptosomes, peroxynitrite or its donor abolished high-affinity choline uptake and synthesis of acetylcholine from acetate, while UA, among other antioxidants, partially prevented these effects [153]. At the same time, there are situations when free radical scavengers, including UA, increase peroxynitrite-induced acetylcholine release and subsequent calcium influx [154]. The aspects of UA activity under conditions of excess peroxynitrite are described in more detail in the table.

## 7 POSITIVE AND NEGATIVE EFFECTS OF URIC ACID ON DIFFERENT TARGETS OF ALZHEIMER'S DISEASE PATHOGENESIS

Mechanisms of interconnection between UA and cognitive function elucidated in the review [18] include oxidative stress (mostly the antioxidant effects of UA, though prooxidant effects are also possible), interaction with β-amyloid (an ambiguous process that should be further investigated, in which both destructive and protective effects of UA were observed, and a time-dependent neuroprotective effect of UA is supposed), inflammation (the crucial role of the TLR4/NF-κB pathway), endothelial dysfunction and vascular damage [18]. The experimentally obtained evidence of the involvement of these and other effects on the beneficial or detrimental effects of UA is summarized in Table **1**.

Data about the influence of UA on fundamental mechanisms such as BDNF and glutamate-induced excitotoxicity were not included in the table because they were obtained from models other than those directly related to AD pathogenesis. Thus, BDNF-related mechanisms were confirmed in SH-SY5Y cells protected by UA from oxidative/nitrosative stress [155]. UA also exerted a beneficial effect on cultured rat hippocampal neurons exposed to glutamate by counteracting oxidative stress, attenuating delayed elevations in intracellular free calcium levels, and preserving mitochondrial function [156].

The interaction of UA with peroxynitrite and its possible inactivation, which was partially described above, is of high interest in the context of oxidative stress. Numerous molecules, for instance, plant phenolic compounds, were shown to activate the endogenous defence mechanisms against oxidative damage, and the hormesis concept was successfully applied to characterize their efficacy in neuroprotection [157, 158]. Moreover, the concept of hormesis as a dose-response phenomenon characterized by low-dose stimulation and a high-dose inhibition explains the role of low molecular endogenous antioxidants (even bilirubin is considered among them) in neurodegeneration [159-161]. One of the basic mechanisms in the efficacy of low doses of such molecules consists in the activation of pro-survival pathways such as heat shock response (HSR) pathways and other ways under the control of protective genes named vitagenes [159].

Of special interest in this context is the role of nitric oxide, which, depending on concentration, is involved in cell death or survival mechanisms in brain cells and is characterized as a “Janus molecule” [160]. Additionally, there is a double-edged role of NO in amyloid-β precursor protein (APP) processing *in vitro*: low (physiological) levels of NO inhibit the amyloidogenic processing of APP, whereas extrahigh (pathological) concentrations of NO favor the amyloidogenic pathway of APP processing, this process is peroxynitrite-related.

As described above, the dependence of UA effects on dose/concentration is established. Notably, UA was also characterized as “two-faced Janus” [162] or a “double-edged sword” [163, 164]. It was stated that the UA molecule exhibits significant physiological activity and can be considered the endogenous signal molecule along with NO, CO, and H_2_S [165, 166]. This similarity, in our opinion, is not accidental and indicates the involvement of the fundamental mechanisms relevant for future research.

## CONCLUSION

Thus, mechanisms associated with uric acid neuroprotective properties represent promising fields for the search of targets in drug discovery as well as in the investigation of Alzheimer’s disease pathogenesis. Changes in uricemia or in uric acid transport between blood and the CNS, including the changes which arise during pharmacotherapy, are important for the prognosis in neurodegenerative diseases (and there are differences between uric acid effects in Alzheimer’s disease and other neurodegenerative disorders).

Proceeding from the analysed clinical studies, the most important modulatory factors for the influence of uric acid on the brain are the presence of gout, sex, cardiovascular risks, low-grade inflammation, education level, and malnutrition. In this context, the most significant and less studied aspects that are promising for further research can be summarized as follows:

1.  Further analysis of the decreased risk of dementia in gout, the establishment of certain pathophysiological mechanisms that are sex-specific and make this association less significant in females, and whether there is a possibility to target these mechanisms pharmacologically.
2.  In-depth research of the different UA role in neuroprotection in males and females.
  •  Since neuroprotective effects of high UA levels are more significant in males, especially if the general population is considered, it is important to determine what mechanisms could be targeted in females to mimic these protective effects or determine the categories of females that benefit most from high uricemia (considering the possible involvement of high uricemia into metabolic syndrome development, especially in postmenopause).
    •  Based on the results of CSF studies within the targeted groups (patients with dementia at different stages of cognitive impairment), it would be rational to investigate the mechanisms of the effect of high uricemia on Aβ1-42 and especially tau, which are specific for females.

3.  Further analysis of the mechanisms of additional effects of diuretics on cognition through the modulation of purine metabolism (studies of the drugs targeting endothelium and able to modulate UA effects or XOD activity are also if great importance).
4.  The possible influence of hyperuricemia as a factor that increases motivation, including to high educational attainment, is of special interest taking into account the protective effect of the latter on cognition in aging (which is known out the context of purine metabolism), thus, mutual enhancement of the positive effect is possible. Nevertheless, it is extremely difficult to identify this interrelationship in modern humans due to hyperuricemia involvement in pathogenesis of the “diseases of civilization”. Clear stratification by educational level, kidney function decline, inflammatory markers, and drugs taken could be helpful in the analysis of the large databases.
5.  General problematic issues in clinical studies, metabolomic analysis, or retrospective data analysis are the need for stratification of patients or examined subjects between AD and other types of dementia, applying criteria of cognitive decline diagnosis as well as stratification by uricemia, data adjustment based on creatinine blood level and intake of drugs influencing purine metabolism. Stratification by low-grade inflammatory markers, malnutrition status, and smoking should obviously be considered in cross-studies. Nevertheless, the unique mechanisms are unlikely to be identified within the continuum of smoking, purine metabolism, and cognition. The reason for this is the extreme complexity of interrelationships between cholinergic effects, endothelial damage, prooxidant-antioxidant disbalance, dysuricemia, *etc*.

Proceeding from the analysed pre-clinical and fundamental studies, the most significant and promising directions for further research can be summarized as follows:

1. The influence on local synthesis, transport, or compartmentalization of UA.
  Given that the biological effects of UA can vary within different biological media, the modulation of the local level of UA within the brain could be further assessed. The enhancement of the local synthesis is not possible for all CNS structures (XOD activity is not ubiquitous there) and also may be problematic due to the increase in prooxidants associated with XOD activity (especially within the vessels). Thus, the most significant is the influence on compartmentalization of UA and its transport within the CNS, for instance, through ITM2B protein (linkage of UA from the gut to the brain is also discussed as a neuroprotective strategy).
2. Interventions at the level of BBB permeability.
  Of significant importance is the question of whether the increased UA levels within the CNS reflect the primary disruption of the BBB or high uricemia can maintain the BBB protective role, for instance, by blocking the penetration of inflammatory cells to the brain (obviously, the situation may vary under the different pathological processes and this also is needed to be clearly classified).
3. Further fundamental research of the role of UA as a possible endogenous signal molecule.
  Its involvement in the enhancement of the fundamental survival mechanisms could be further characterized (since it is dose/concentration dependent, the hormesis hypothesis can be applied in this context).
4. Further fundamental studies of the links between neurodegeneration and such evolutionarily ancient mechanisms as electrolyte exchange (both of these processes being interrelated with purine metabolism).
  The certain promising task within this fundamental problem is clarification of the role of UA within the cerebral vascular bed under the conditions of excess sodium - are there differences with other vascular pools (given that the negative role of UA is clearly established in metabolic syndrome)? Among the connection points, there is NO availability (given that UA has multi-directional effects on NO - it can inactivate free radicals and contribute to NO bioavailability or deplete NO levels) or, possibly, neurohumoral responses primarily induced by sodium excess (since UA is also the factor involved in salt hypersensitivity and sodium retention).
5. Targeting of the certain links between purine metabolism and AD-related hypometabolism.
  Such processes could be addressed as:
  •  Possibilities of modulations of glucose supply to the brain and its utilization.
  •  An interrelationship between creatine/creatinine energy cycle in the brain as well as in muscle and amyloid physiology.
6. Establishing the fundamental differences between UA's role in AD and in vascular dementia.
  Since the effects of high UA concentrations in the neurons and within the vessel wall differ, it is important to determine what links of the antioxidant system can be differentially targeted by UA in AD and in vascular dementia, causing the negative role of UA in the latter. Evaluation of the interplay between these prooxidant-antioxidant processes and lipid metabolism, namely the involvement of HDL, is also of high importance.
7. Further studies of the proinflammatory potential of UA, especially significant for patients with gout.
  Establishing the conditions in which activation of Toll-like receptor 4 (TLR4)/NF-κB pathway and NLRP3 inflammasome mediates the neurotoxic response and whether it is possible to influence these pathways maintaining the neuroprotective role of UA or to determine the categories of subjects that have a lower risk of the proinflammatory and neurotoxic response and thus benefit most from high uricemia.
8. Further studies of XOD involvement into neurodegenerative processes and potential of its inhibitors.
  •  Obtaining additional data on biochemical markers of XOD involvement to neurotoxicity through oxidative stress enhancement (especially within the vascular bed in the CNS, its interrelationship with cathepsin B and nexin-1 activity) as well as possible ways to counteract these processes.
  •  Further differentiation between competitive and non-competitive XOD inhibitors effects (since distinct data were obtained for allopurinol and febuxostat).
9. Establishing the fundamental mechanisms causing sex-specific UA influence on neurodegeneration.
  The most promising processes are as follows:
  •  Influence of androgens/estrogens or other sex-related biochemical factors on the interplay between pathogenic proteins and prooxidants.
  •  Modulation of estrogens' fundamental neuroprotective effects by UA (especially of the influence of estrogens on neuronal plasticity or astrocytes gap junctions).
  •  Modulation of the vasoactive effects of estrogens by UA (since the negative effects of UA are clearly seen in vascular dementia).
10. Interrelationship between UA effects and the cholinergic system.
  •  Evaluation of the mechanism of UA antioxidant and cytoprotective activity within cholinergic synapses and possibilities of targeting these mechanisms by other related compounds.
  •  The search for polymodal compounds targeting both purinergic and cholinergic mechanisms.

## Figures and Tables

**Table 1:** The experimentally obtained evidence on the beneficial or detrimental effects of uric acid on the different targets associated with dementia development.

**The Effects Associated with Amyloid-β**							
***In vitro***							
**Beneficial**							
**Cell Line**		**Uric Acid Concentration**	**Results**				**References**
SHSY5Y neuroblastoma cells stably expressing wild-type 695-amino acid isoform of the amyloid-β precursor protein (APP) treated with sodium nitroprusside		10 mM	Reversal of the amyloidogenic activity of sodium nitroprusside (action of UA as a peroxynitrite scavenger)				[167]
Mouse microglia BV2 cells exposed to oligomer Aβ		Concentrations up to 400 µM	Protection of BV2 cells from Aβ toxicity (UA at a concentration of 100 µM) by upregulating the expressions of Beclin-1, LAMP1, and the LC3II/I ratio, inhibition of TFEB abolishes these effects				[89]
Primary hippocampal neurons from presenilin-1-mutant knock-in mice exposed to Aβ_1-42_		200 µM, Pretreatment for 1 h	Increased cells survival through peroxynitrite scavenging and oxidative stress counteraction				[168]
**Detrimental**							
SHSY5Y neuroblastoma cells incubated with amyloid β		40 µM or higher (close to those achievable in cerebrospinal fluid in mild hyperuricemia)	Decrease in cell viability and potentiation of the proapoptotic effect of amyloid β, increased formation of 4-hydroxynonenal (prooxidant marker) and the expression of PPARβ/δ				[169]
***In vivo***							
**Beneficial**							
**Model**		**Uric Acid Dose or Intervention**	**Results**				**References**
Infusion of Aβ1-40 into the brain ventricle of Wistar rats		200 mg/kg i.p. daily for 5 days after the start of infusion	Prevention of tyrosine nitration of synaptophysin and the impairment of nicotine-evoked ACh release induced by Aβ (through peroxynitrite scavenging by UA)				[170]
1) infusion of Aβ1-42 into the brain ventricle of C57BL/6 J mice; 2) APP23/PS45 double transgenic mice (behavioral tests at the age of 5 months)		2) oxonic acid (100 mg/kg/day i.p.) and inosine (200 mg/kg/day i.p.) for 3 weeks	Significant alleviation of memory decline in both models. UA activated microglia and upregulated the autophagy-related proteins such as LC3II/I ratio, Beclin-1, and LAMP1 in the hippocampus. The improvement in cognitive function is associated with activation of TFEB to induce microglia autophagy and facilitate Aβ degradation				[89]
**Detrimental**							
Sprague-Dawley rats		Diet containing 1% of yeast and 0.025% potassium oxonate *per se* or combined for 48 weeks	Cognitive impairment signs in Morris water maze test, impaired prooxidant and inflammatory markers (SOD, MDA, TNF-α, but not GSH-Px and IL-1β) and increased level of β-amyloid peptide in hippocampus. The detrimental effects were dependent on serum UA concentration				[171]
**Clinical Studies**							
**Beneficial**							
**Category of Patients**		**Main Indicators**		**Results**			**References**
Mild cognitive impairment or AD (mean age 73,7 years, males and females, data from the ADNI database, mean duration of serial studies 2.9 years)		CSF Aβ_1-42_, tau, total tau (t-tau) and phospho-tau (p-tau), uricemia, mini-mental state examination, AD Assessment Scale - Cognitive Subscale		Protective effects of UA on the longitudinal cognitive decline independent of and interactively with CSF AD biomarkers, including the alleviation of the detrimental effect of Aβ_1-42_ on cognitive decline or tau on cognitive decline (especially significant in the mild cognitive impairment and dementia subgroups, and in females)			[53]
Chinese cohort of patients, mild cognitive impairment (median age 66), dementia (median age 68, median age in cognitively unimpaired control was 41)		CSF and serum Aβ_40_, Aβ_42_, total tau (T-tau) and CSF phosphorylated tau-181 (P-tau181), neurofilament light chain, mini-mental state examination		Serum UA levels first increased and then decreased, and the peak was in the stage of mild cognitive impairment of AD. UA (as well as Aβ42) alleviated the detrimental effects of amyloid/tangle/ neuro-degeneration biomarkers on cognitive function and Aβ42 on tau. Positive correlations of serum UA with mini-mental state examination and with plasma A-β_42_. Higher levels of serum UA weakened the correlation of MMSE scores with CSF amyloid/ tangle/neurodegeneration markers and the correlation of CSF Aβ42 with tau			[172]
**Detrimental**							
**Category of Patients**		**Main Indicators**		**Results**			**References**
Preclinical AD, cognitively intact individuals with or without tau pathology and neurodegeneration, mean age 62, males and females, Chinese cohort of patients		CSF Aβ_1-42_, Aβ_1-42_/Aβ_1-40_, p-Tau/Aβ_1-42_, and t-Tau/Aβ_1-42_; mini-mental state examination		Higher burden of cerebral amyloidosis in the patients with the highest (> 75^th^ percentile) serum UA when compared to lowest serum UA			[173]
**Note**. The first increasing and then falling trend of sUA levels could be due to the early compensatory antioxidative function for Aβ42 and the later decompensation and self-exhaustion for anti-Aβ42 and the Aβ-tau pathological process or other amplifying vicious cascade in the later stage of AD. Therefore, serum UA could be a protective factor of cognitive impairment, and this effect predominantly acted on dementia and AD stage [172]. In this context, the difference between studies of Huang *et al*. (2022) and that of Li *et al*. (2021) can be due to the time of sampling - in the patients cognitively intact still with the amyloid/tangle/neurodegeneration biomarkers increase in serum UA could have been a compensatory reaction still not able to decelerate the pathological process significantly.							

**Table:** 

**The Effects on the Cerebral Amyloid Angiopathy** The data about UA role in cerebral amyloid angiopathy were also considered, since the shared role of Aβ deposition is emphasized in this state as well as in AD (characterized as a crosstalk between neurodegenerative and cerebrovascular pathology) [174]								
***In vitro***								
**Beneficial**								
**Cell Line**	**Uric Acid Concentration**			**Results**			**References**	
Human brain microvascular endothelial cells (HBMEC), human vascular smooth muscle cells (HBVSMCs)	237 µM			Reduction of the negative effects of Aβ on the vascular endothelium and smooth muscle cells and protection of the vascular wall, namely, the tight junction integrity *in vitro* (the markers were occludin and ZO-1 (tight junction protein-1))			[175]	
***In vivo***								
**Beneficial**								
**Model**	**Uric Acid Dose or Intervention**			**Results**			**References**	
APP23 transgenic mice (extensive β-amyloid pathology)	UA at a dose of 62.5 mg/kg or allopurinol at doses of 5 mg/kg up to 10 mg/kg			Serum UA (124 µM in APP23 mice *vs.* 169 µM in WT controls, 197 µM in UA group and 76 µM in allopurinol group) positively correlated with the integrity of blood vessel wall and negatively correlated with the expression level of Ab_1-42_ and the degree of amyloid deposition in blood vessels (protective effect of UA on the maintenance of tight junctions of the vascular endothelium). Further increase in Aβ_1-40_ and Aβ_1-42_, decrease in ZO-1 and occludin expression levels in hypouricemic mice			[175]	
**Clinical Studies**								
**Beneficial**								
**Category of Patients**	**Main Indicators**			**Results**			**References**	
Patients with the cerebral amyloid angiopathy(CAA)-associated intracerebral hemorrhage	Uricemia, the total hemorrhagic lesions			Decreased uricemia in patients with possible CAA and further decrease in patients with probable CAA, uricemia potentially reflected the severity of Aβ deposition. No associations between uricemia and the distribution of hemorrhagic lesion, as well as neurological impairment			[176]	
**The Effects Associated with Proinflammatory Markers**								
***In vitro***								
**Detrimental**								
**Cell Line**	**Uric Acid Concentration**			**Results**	**References**			
Primary cultured hippocampal cells from rats embryonic brains	600 ng/ml for 24 and 48 h			Activation of the NF-κB pathway; p65/RelA translocation to the nucleus was markedly increased, mRNA levels of proinflammatory cytokines (namely, *Nfkbia, Il6, Il1b, Ikbkb, Ccl2, and Tlr4*) were significantly increased	[31]			
***In vivo***								
**Detrimental**								
**Model**	**Uric Acid Dose or Intervention**			**Results**	**References**			
1) male Wistar rats (7-8 weeks old) 2) TLR4 knock-out mice, (TLR4−/−) C57BL/6 strain	1) high-UA diet (2% of oxonic acid (OA) and 2% of UA from 1 day to 12 weeks)			Increased expression of proinflammatory cytokines, activation of the Toll-like receptor 4 (TLR4)/ NF-κB pathway, increases gliosis in rat hippocampus. Hippocampal inflammation and memory deficits in C57BL/6 mice realized *via* the TLR4/NF-κB pathway	[31]			
1) Sprague-Dawley rats (11-12 weeks old); 2) NF-κB p50-knockout male mice (B6129PF2/J)	1) high-UA diet (2% of OA and 2% of UA, 12 weeks) 2) 2 μl UA (600 ng/ml) intracerebro-ventricularly for 3 weeks			Decreased cognitive abilities in the Morris water maze, increased UA level in hippocampus tissue including mitochondria, decrease in ATP production and cytochrome c oxidase activity The mechanism is realized through triggering of intramitochondrial NF-κB inhibitor α (IκBα)/ NF-κB pathway to downregulate the subunit III of cytochrome c oxidase	[90]			
**Clinical studies**								
**Detrimental**								
**Category of patients**	**Main Indicators**			**Results**	**References**			
Patients with AD (mean age 76 years, mean age of the control subjects 71 years, males and females)	Uricemia, blood routine biochemistry, screening for MTHFR 1298A->C mutation			Uricemia was an independent predictor of AD, a positive interaction of UA with homocysteine (which was also an independent predictor of AD)	[61]			
Patients which underwent magnetic resonance imaging with quality coronal views of the hippocampus and the absence of abnormalities that might confound interpretation of the links with uricemia	Uricemia and magnetic resonance imaging			Hyperintense signaling in the hippocampus as a possible sign of gliosis (there were certain limitations since only a limited area of the tissue was examined and similar changes of the signaling could be caused by inflammatory edema, inflammation, hyperplasia, and tumors), increased density of both astrocytes and microglia	[31]			

**Table:** 

**The Effects Associated with Peroxynitrite Scavenging** (the data on peroxynitrite associated with the direct measurement of β-amyloid and tau proteins are given above)								
***In vitro***								
**Beneficial**								
**Cell Line**	**Uric Acid Concentration**			**Results**	**References**			
PC12 pheochromocytoma cells expressing cholinergic phenotype, exposed to peroxynitrite generator 3-morpholino-sydnonmine (SIN-1)	No data			UA as a peroxynitrite scavenger attenuated the inhibitory actions of SIN-1 on nerve growth factor (NGF)/TrkA signaling (peroxynitrite-induced cytotoxicity is directed to tyrosine residues of tyrosine kinase receptors that are crucial for the maintenance of cholinergic neurons and undergo nitration in this case)	[177]			
Primary cultured hippocampal cells from the embryonic brains of rats, apoptosis induction by by FeSO_4_ and Aβ_25-35_	1-100 µM			Increase in the cells survival (dependent on concentration and maximal at a concentration of 100 µM), suppression of the increases in neuronal peroxynitrite levels. The effect was maintained at a presence of activator or blocker of NF-kB activation	[178]			
Pheochromocytoma PC6-V and PC6-MnSOD cells, apoptosis induction by FeSO_4_, Aβ_25-35_, sodium nitroprusside	200 µM			Apoptosis prevention, increase in cells survival in all of the modes of apoptosis used. Prevention of sodium nitroprusside-induced mitochondrial alterations. UA protective activity is linked to peroxynitrite scavenging	[179]			
***In vivo***								
**Beneficial**								
**Model**	**Uric Acid Dose or Intervention**			**Results**		**References**		
Bilateral injection of peroxynitrite donor SIN-1 into the hippocampus of rat brain	UA infusion (15 µl, 5 mM) into the left ventricle 1 h before SIN-1 injection (25 mM)			Prevention of the increase in the level of nitrated and hyperphosphorylated tau in the hippocampus		[180]		
Wistar rats, infusion of Aβ1_1-40_ into the right cerebral ventricle for 5 or 10 days	200 mg/kg, i.p. daily for 5 days or 10 days after the start of Aβ infusion			Prevention of tyrosine nitration of synaptophysin as well as of the impairment of nicotine-evoked ACh release induced by Aβ1_1-40_		[170]		
**The Effects Associated with the Other Aspects of Antioxidant-Prooxidant Status** (only the data directly related to the AD pathogenesis were included, the results concerning peroxynitrite scavenging are described above)								
***In vitro***								
**Beneficial**								
**Cell Line or Model**	**Uric Acid Concentration**			**Results**		**References**		
Antioxidant experiments with saline solutions modelling CSF	30 µM and 357 µM (0.5 and 6.0 mg/dl)			Significant inhibition (lower concentration) and complete inhibition (higher concentration) of the oxidative changes caused by reactions of 20 µM hydrogen peroxide with free bivalent iron at 9.8 µM and at a low hemoglobin level of 12 mg/dl of saline. UA levels attainable in blood plasma were used and it was concluded that supplementation with UA metabolic precursor inosine (similarly to ascorbic acid) has the potential for preventing or attenuating the progression of AD		[181]		
***Clinical Studies***								
**Beneficial**								
**Category of Patients**	**Main Indicators**			**Results**		**References**		
Patients with AD (mild, moderate, severe, mean age at a range of 74-78 years, males and females)	Uricemia, serum levels of cerulo-plasmin, C-reactive protein, homo-cysteine, copper, iron, and zinc			Decreased level of UA and ceruloplasmin in patients with AD, positive correlation between uricemia and Mini-Mental State Examination scores. UA was considered as a possible risk factors that determine the severity of AD		[182]		
Patients with mixed Alzheimer disease/vascular dementia, median age of 75 years, males and females	Uricemia, products of oxidative DNA damage repair (8-oxo-2'-deoxyguanosine and 8-oxoguanine), antioxidant vitamins in blood plasma			Significant decrease of uricemia together with increased urinary excretion of oxidative DNA damage repair products and the level of 8-oxoguanine in CSF, reduced levels of ascorbic acid and retinol in plasma. These shifts were considered as the prerequisite for antioxidants efficacy for the inhibition of dementia progression		[183]		

## LIST OF ABBREVIATIONS

AD: Alzheimer’s Disease
ADNI: Alzheimer’s Disease Neuroimaging Initiative
APOE4: Apolipoprotein E4
APP: Amyloid-β Precursor Protein
Aβ: Amyloid beta
BBB: Blood-brain Barrier
CNS: Central Nervous System
CSF: Cerebrospinal Fluid
eGFR: Estimated Glomerular Filtration Rate
HDL: High-density Lipoproteins
NF-κB: Nuclear Factor-kappa B NLRP3
inflammasome: NLR Family Pyrin Domain Containing 3 Inflammasome
OA: Oxonic Acid
SNPs: Single Nucleotide Polymorphisms
TLR: Toll-like Receptor
UA: Uric Acid
XOD: Xanthine Oxidase
